# Chronic exposure to imidacloprid or thiamethoxam neonicotinoid causes oxidative damages and alters carotenoid-retinoid levels in caged honey bees (*Apis mellifera*)

**DOI:** 10.1038/s41598-018-34625-y

**Published:** 2018-11-02

**Authors:** Maxime Gauthier, Philippe Aras, Joanne Paquin, Monique Boily

**Affiliations:** 1 Département des Sciences Biologiques, Université du Québec à Montréal, C.P. 8888, Succursale Centre-Ville, Montréal, Québec, H3C 3P8 Canada; 2 Département de Chimie, Université du Québec à Montréal, C.P. 8888, Succursale Centre-Ville, Québec, H3C 3P8 Canada

## Abstract

Over the last decade, the persistent dwindling of the populations of honey bees has become a growing concern. While this phenomenon is partly attributed to neonicotinoids (NEOCs), chronic exposures to these insecticides at environmentally-relevant concentrations are needed to fully estimate their implications. In this study, honey bees were orally exposed for 10 days to low field-realistic concentrations of NEOCs known for their effects on the cholinergic system (imidacloprid – IMI or thiamethoxam – THM). Selected biomarkers were measured such as acetylcholinesterase (AChE) activity, lipid peroxidation (LPO), α-tocopherol as well as several forms of vitamin A (retinoids) and carotenoids. Bees exposed to IMI showed lower levels of two carotenoids (α-carotene and α-cryptoxanthin) and α-tocopherol. The THM exposure increased the oxidized vitamin A metabolites in bees conjointly with the LPO. These results could be the consequence of a pro-oxidant effect of NEOCs and were observed at levels where no effects were recorded for AChE activity. This study reveals that exposure to low levels of NEOCs alters the carotenoid-retinoid system in honey bees. This would merit further investigation as these compounds are important in various aspects of bees’ health. Overall, this study contributes to the development of biomonitoring tools for the health of bees and other pollinators.

## Introduction

Global honey bee loss has become a prior economic and environmental concern. While there is a consensus that this situation is the result of several factors, abnormal bee deaths have been associated with the widespread use of neonicotinoids (NEOCs). Although several countries restricted the use of NEOCs in order to protect pollinators (http://europa.eu/rapid/press-release_IP-13-708_en.htm, URL verified on October 25, 2018), the underlying biochemical derangements associated with chronic exposure should be better documented, as these insecticides are still widely used in North America.

Commercialized in the 1990 s as systemic and broad-spectrum insecticides, NEOCs are intended for agricultural pest management. Given their selective toxicity for insects and their wide array of applications (e.g., seed coating, foliar spray and soil drenches), they became the most widely used class of insecticides and now account for more than 23.7% of the world market share^1^. In the last decade, the proportion of land cultivated with NEOCs-coated seeds, especially with imidacloprid (IMI), thiamethoxam (THM) and clothianidin, has greatly increased in North America. In 2012, almost the entirety of Canada’s main oleaginous culture, canola, most of which is grown in Manitoba and Saskatchewan, was treated with NEOCs coating^2^. In addition, the dominant culture in eastern Canada (Quebec and Ontario) are comprised of NEOCs-treated corn and soy (99% and 30–65% respectively)^3^ (http://www.omafra.gov.on.ca/english/about/beehealthpresentations/omafcrop.htm, URL verified on October 25, 2018).

Levels of NEOCs in agroecosystems may vary depending on modes of application and climatic factors. Several studies reported NEOCs in bee-relevant matrices (pollen, nectar, honey and beeswax) in which concentrations were up to 200 µg/kg^4^. Moreover, NEOCs residues were found in honey bee tissues, confirming the exposure of bees to these insecticides through their foraging activities. Foragers may also get in contact with particles of these chemicals via dust or guttation droplets emitted during sowing or application^5^.

NEOCs were designed to act as insect-specific acetylcholine agonists by binding to the postsynaptic nicotinic acetylcholine receptors (nAChRs) in the central nervous system, thus causing paralysis and ultimately death of the crop pests. When honey bees were fed with technical or commercial NEOCs formulations, observed side effects were reported on foraging activity^6^, memory^7^, immunity^8^, reproduction^9^ and development^10^. There is a limited understanding of the mechanistic establishment of these derangements in the honey bee. However, biochemical abnormalities have been reported following exposure to NEOCs and may be linked to the previously mentioned disorders. In bees, increases in the acetylcholinesterase activity have been observed following exposure to NEOCs revealing its potential interest as a biomarker^11,12^. Also THM promotes oxidative stress in honey bees as shown by the increased activity of catalase and glutathione S-transferase^13^. Oxidative stress occurs when the antioxidant defenses of an organism are unable to counterbalance the oxidant insults (free radicals) and may consequently alter several cell constituents (lipids, DNA and proteins) leading to tissue and organ function disorders. To maintain a redox homeostasis, *A. mellifera* relies on a battery of enzymatic and non-enzymatic antioxidants, including diet-derived antioxidants such as tocopherols (vitamin E) and carotenoids^14,15^.

**Figure 1:**
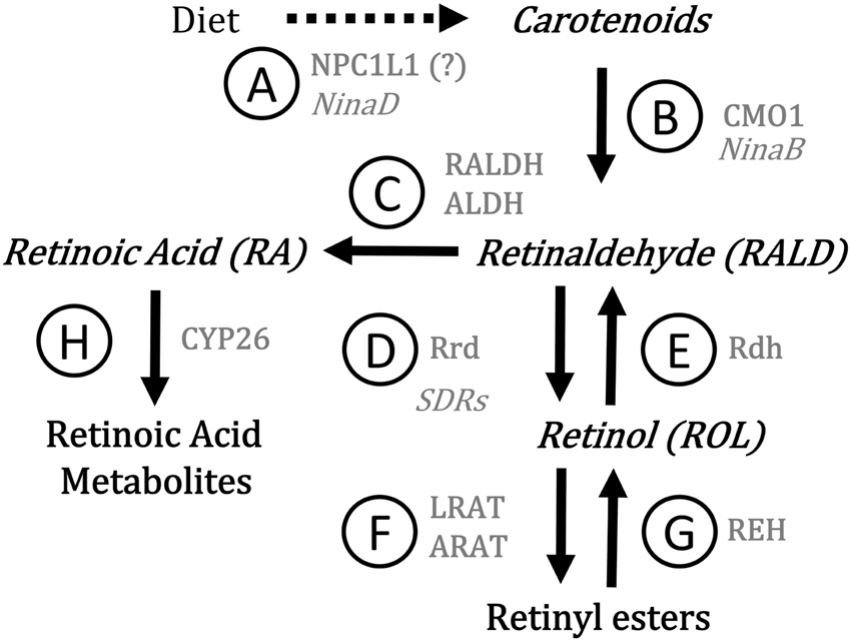
Overview of carotenoid-retinoid metabolism. The name of the proteins mediating the different steps in vertebrates are in grey roman letter while their equivalents in insects, when known, are in grey italics. (**A**) NPC1L1 (Niemann–Pick C1-like 1)/NinaD (Neither inactivation nor afterpotential D); (**B**) CMO1 (Carotenoid mono-oxygenase 1)/NinaB (Neither inactivation nor afterpotential B); (**C**) RALDH (Retinal dehydrogenase) or ALDH (Aldehyde dehydrogenase); (**D**) Rrd (Retinal reductase/SDRs (Short chain dehydrogenases/reductases); (**E**) Rdh (Retinol dehydrogenase); (**F**) LRAT (Lecithin retinol acyltransferase) or ARAT (Acyl CoA:retinol acyltransferase; (**G**) REH (Retinyl ester hydrolase); (**H**) CYP26 (Cytochrome P450 26).

In addition to their antioxidant role, carotenoids are precursors of retinoid compounds including retinol (ROL), retinaldehyde (RALD) and retinoic acid (RA) (Fig. 1). In vertebrates, the functions of carotenoids and retinoids are extensively studied because of the implication of RA in various crucial processes during embryonic development (e.g., axial/regional patterning, organogenesis, limb development and neurogenesis) and throughout adulthood (e.g., vision, organ/tissue homeostasis and regeneration). Levels of RA must be tightly controlled to regulate these processes as RA binds to members of the RAR/RXR/PPAR nuclear receptor family to modulate gene expression. As depicted in Fig. 1, CYP450 enzymes are responsible for oxidation of RA producing “oxo” metabolites (13-*cis*-4-oxo-RA and all-*trans*-4-oxo-RA). Regulation of RA transcriptional activity also involves isomerization between “*trans*” and “*cis*” forms which bind with differential affinity to the nuclear receptors. In insects, retinoids have been mostly studied relatively to their role as chromophores in vision. However, recent literature suggests that retinoids, namely RA, would play a similar developmental role in insects and in vertebrates (see review by Albalat 2009^16^). In fact, studies showed that unbalanced RA leads to developmental malformations in the tick (*Rhodnius prolixus*)^17^ and *Drosophila melanogaster*^18^ and influences metamorphosis and organogenesis in the firebug (*Pyrrhocoris apterus*), the red cotton stainer (*Decipifus cingulatus*) and the mealworm (*Tenebrio molitor*)^19^. Also, the 9-*cis*-RA isomer had a positive effect on cells isolated from *Locusta migratoria* and stimulated neurite outgrowth^20^. Retinoid metabolism has been shown to be sensitive to agrochemicals in fishes, frogs, birds and mammals (see review by Boily *et al*. 2004^21^). To our knowledge, the present paper is the first to investigate the NEOCs-induced alterations in the honey bee’s carotenoid-retinoid metabolism under laboratory controlled conditions.

So far, toxicological studies involving bees exposed to sub-lethal doses of NEOCs were mostly conducted for short periods of time. Effects of longer exposures to low and environmentally realistic concentrations are poorly documented. Taking into account the generally degrading health status of pollinators, the harmful influence of NEOCs on the honey bee, and the lack of knowledge regarding the biological functions of the carotenoid/retinoid metabolism in these insects, the goal of this study was to further document biochemical alterations caused by a chronic exposure of honey bees to environmentally realistic concentrations of the NEOCs. In this paper, we evaluated the AChE activity as a potential biomarker for NEOCs exposure in the honey bee. Because of the pro-oxidative effects of NEOCs, we believe that they could interfere with the retinoid metabolism. We therefore estimated the capacity of NEOCs to cause lipid peroxidation (LPO) or alter the levels of retinoids and lipophilic antioxidants such as α-tocopherol as well as carotenoids. Based on the tight biochemical regulation of the retinoid machinery and its sensitivity to agricultural contaminants, we posit that the retinoid metabolism could be used for the development of retinoid-based biomarkers to assess the health of bees as well as other insects.

## Results and Discussion

Honey bees are exposed to NEOCs mainly through pollen, nectar and guttation fluids. The exact extent of the exposure is not known but concentrations of NEOCs around 1 µg/kg fresh weight (about 1.5 ng/bee) have been encountered in bees from some European countries and from the United States^22^. These levels are in the range of acute oral LD_50_ (around 5 ng/bee)^12^. In the present study, bees were exposed on a daily basis to low levels (<3% of LD_50_) of IMI or THM-contaminated syrups for 10 days. Estimated daily intake ranged from 2 to 81 pg/bee for IMI and 4 to 124 pg/bee for THM (Table 1). There was no statistical difference in daily volume of syrup intake, indicating the non-repellent character of the syrups. Under these conditions, IMI or THM did not cause dose-dependent mortality. In fact, the mean survival for all groups of bees was between 95 and 100% (Table 1). A significant difference with the control group was found only with the concentration 120 ng/100 ml of THM (χ2 = 4.56, *df* = 1, *p* < 0.05, Log-rank test), although survival was still at 95.1%. In 2016, the Organisation for Economic Co-operation and Development^23^ stipulated that mortality for chronic oral toxicity tests should not be more than 15%^23^. The mortality of less than 5% observed in our chronic exposures fulfilled this guideline as well as our own objective of measuring biochemical indicators in bees exposed to non-lethal NEOCs levels.

**Table 1:** Daily NEOC intake, survival and body mass for honey bees exposed to IMI and THM for 10 days.

NEOC (ng/100 ml syrup)	Daily syrup intake (μl/bee)	Daily NEOC-intake (pg/bee)^a^	Survival (%)	Body mass (g)^b^
*IMI*				
0 (Control)	39.2 ± 7.0	0.0	94.8 ± 7.0	1.46 ± 0.06
6	33.8 ± 3.4	2.1	96.4 ± 5.4	1.43 ± 0.07
20	39.2 ± 1.1	7.9	98.6 ± 2.8	1.44 ± 0.05
60	39.1 ± 1.2	25.2	94.9 ± 4.8	1.51 ± 0.05
200	39.2 ± 5.5	81.0	97.9 ± 2.7	1.45 ± 0.06
*THM*				
0 (Control)	32.4 ± 3.3	0.0	99.3 ± 1.4	1.43 ± 0.07
12	32.6 ± 4.3	4.1	98.6 ± 2.9	1.45 ± 0.04
40	32.7 ± 6.7	12.9	97.2 ± 2.2	1.40 ± 0.09
120	32.0 ± 4.3	42.0	95.1 ± 2.7*	1.41 ± 0.06
400	29.5 ± 3.1	124.3	100 ± 0.0	1.40 ± 0.06

^a^Estimated values: NEOC concentration in syrup (pg/μl) x daily syrup intake (μl/bee) (see Materials and methods section for details).

^b^Estimated values for 10 bees.

Values (means ± SD) are for 4 replicates (cages) per concentration (≈120 bees). *p < 0.05 Log-rank (Mantel-Cox) test.

Table 2 shows no significant alteration of the head protein concentration or of AChE activity in bees fed with syrups containing up to 200 ng/100 ml of IMI or 400 ng/100 ml of THM. So far, for insects, few studies investigated the NEOC-induced effects on the activity of AChE. Morakchi *et al*. (2005)^24^ orally exposed newly emerged males and females *Blatella germanica* to acetamiprid (2% of LD_50_ for 24, 48 and 72 hours) and reported an augmentation in AChE activity over time for control individuals of both sexes and a diminution in AChE activity in females at every endpoint. Azevedo-Pereira *et al*. (2011)^25^ exposed for 96 h 10-day old third instar larvae of *Chironomus riparius* to water containing various concentrations of a commercial formulation of IMI (Confidor 200 SL) (0, 0.5, 1.5 and 3.0 mg of IMI L^−1^) and reported a diminution of AChE activity upon the IMI exposure. These contrasting results were obtained with important differences in methodology (duration of the exposure, insect species, developmental stages and insecticide). This makes the comparison with our study challenging. However, in a study conducted by Boily *et al*. (2013)^12^, adult bees exposed for 10 days to an IMI-contaminated syrup (80 to 300 pg/bee) showed increases in AChE activity and hyperactivity behavior at all doses tested. The present study investigated a spectrum of lower doses with a technical IMI formulation while Boily *et al*. (2013)^12^ used a commercial formulation (Admire 240 F®). Commercial formulations are known to include additives, which may explain why Boily *et al*. (2013)^12^ observed an increased AChE activity at 80 pg/bee while we measured no effect at the same dose. A greater toxicity of the commercial formulations of IMI has been reported in other models: human cells^26^, green frog^27^ and *Daphnia magna*^28^. Our results indicate that the ingested amounts of IMI were below those producing biological effects on AChE activity.

**Table 2:** Means ± SD for proteins, AChE activity, carotenoids, α-tocopherol and triglycerides in bees exposed to IMI and THM for 10 days.

NEOC (ng/100 ml syrup)	Proteins (mg/g tissue)	AChE (mOD/g prot/h)	α-Carotene (ng/g tissue)	β-Carotene (ng/g tissue)	α-Cryptoxanthin (ng/g tissue)	β-Cryptoxanthin (ng/g tissue)	Lutein (ng/g tissue)	Zeaxanthin (ng/g tissue)	α-Tocopherol (ng/g tissue)	Triglycerides (ng/g tissue)
*IMI*										
0 (Control)	32.2 ± 6.5	0.51 ± 0.21	254 ± 13	116 ± 44	83.6 ± 16.7	66.2 ± 10.3	159 ± 26	141 ± 17.5	175 ± 19	495 ± 112
6	36.2 ± 6.8	0.43 ± 0.14	263 ± 23	124 ± 39	80.8 ± 8.60	68.5 ± 6.90	158 ± 11	140 ± 16.0	166 ± 19	502 ± 920
20	30.5 ± 8.5	0.51 ± 0.21	251 ± 37	121 ± 45	79.2 ± 15.9	68.5 ± 7.90	157 ± 22	136 ± 16.5	164 ± 30	441 ± 600
60	34.9 ± 11.8	0.51 ± 0.21	212 ± 21*	078 ± 24	71.4 ± 10.9	60.1 ± 6.80	138 ± 12	123 ± 13.7	148 ± 23	486 ± 101
200	33.2 ± 8.2	0.43 ± 0.13	233 ± 21^Δ^	119 ± 43	73.5 ± 8.8^Δ^	67.8 ± 8.90	155 ± 24	141 ± 19.6	158 ± 27^Δ^	509 ± 108
*THM*										
0 (Control)	37.8 ± 8.5	0.47 ± 0.17	162 ± 50	199 ± 73	77.5 ± 19.8	41.3 ± 7.00	206 ± 43	93.8 ± 22.8	129 ± 32	714 ± 780
12	39.9 ± 9.9	0.39 ± 0.13	167 ± 49	132 ± 46	63.9 ± 11.5	38.8 ± 9.70	175 ± 56	88.4 ± 18.2	134 ± 21	682 ± 850
40	38.7 ± 7.4	0.38 ± 0.19	140 ± 28	203 ± 49	73.6 ± 12.4	38.2 ± 4.80	168 ± 42	73.3 ± 19.3	132 ± 43	692 ± 860
120	36.2 ± 6.5	0.44 ± 0.16	169 ± 39	241 ± 85	72.2 ± 17.1	41.4 ± 6.80	195 ± 43	87.9 ± 16.0	153 ± 36	673 ± 127
400	34.9 ± 11.9	0.42 ± 0.18	180 ± 38	161 ± 35	77.6 ± 14.0	41.1 ± 3.70	174 ± 35	78.1 ± 13.2	137 ± 21	661 ± 115

Values are for 10 pools of 4 bee heads for proteins and AChE activity and 8 pools of 10 whole bees for carotenoids, α-tocopherol and triglycerides. *p < 0.05 GLM one-way analysis of variance followed by Dunnett *t* test, and ^Δ^*p* < 0.05 Jonkheere-Terpstra trend test.

Carotenoids are lipophilic pigments having antioxidant activity due to their scavenging capacity of reactive oxygen species^29^. In our study, bees exposed to THM did not exhibit any significant alterations in carotenoid contents (Table 2). Following IMI exposure, β-carotene, β-cryptoxanthin, lutein and zeaxanthin levels did not differ significantly from the control values. However, exposure to 60 ng/100 ml IMI caused a significant diminution in α-carotene contents compared to the control group (*F*_4,33_ = 4.62, *p* < 0.01) (Table 2). Moreover, with the augmentation of IMI exposure levels, a significant negative Jonkheere-Terpstra test (used to postulate on the dose-dependency of a response considering the augmentation of an independent variable) was obtained for α-carotene (*JT* = 18.0; *p* < 0.05; n = 38) and α-cryptoxanthin (*JT* = 45.0; *p* < 0.05; n = 37) (Table 2). The effects of IMI on carotenoids have been reported for birds. When fed a diet of seeds coated with a commercial formulation of IMI (Escocets®), red-legged partridges (*Alectoris rufa*) showed a modified beak tint and reduced eye-ring pigmentation, both of which are carotenoid-based colorations^30^. The authors suspected an IMI-induced oxidative metabolism of zeaxanthin leading to a more important formation of asthaxanthin. In the present study, altered carotenoid levels could be due to a decreased absorption in the midgut through alteration of a NinaD receptor. A similar class B scavenger receptor is expressed in *D. melanogaster* and was shown to be essential for carotenoid uptake^31^.

**Figure 2:**
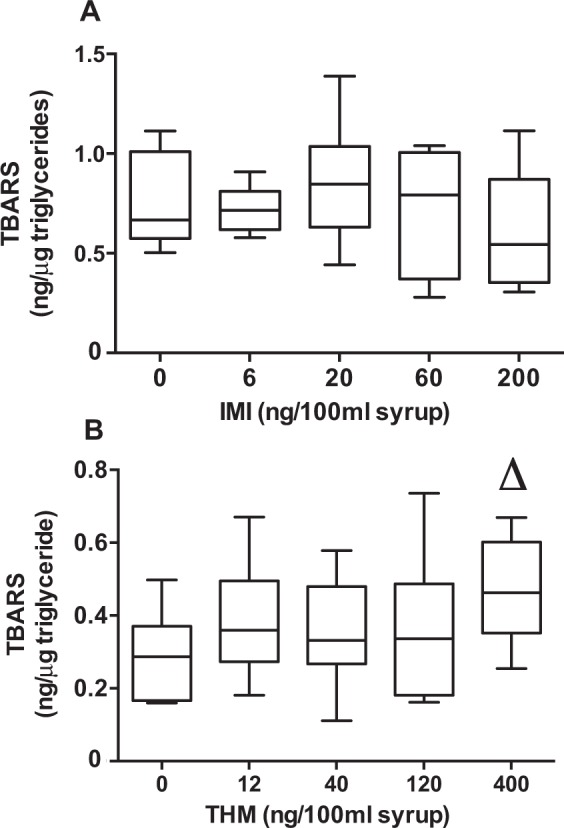
TBARS measured in honeybees exposed to increasing concentrations of IMI (**A**) and THM (**B**). Boxes (8 pools of 10 bees) extend from 25th to 75th percentile; line is the median and whiskers show the largest to smallest observed values. Δ*p* < 0.05 Jonkheere-Terpstra trend test.

Imidacloprid exposure has been reported to induce oxidative stress, notably the LPO, in various organisms including plants^32^, invertebrates^33^, fishes^34^, birds^30^ and rats^35,36^. A similar response was expected in honey bees, yet no increase in LPO was detected after exposure to syrups containing up to 200 ng/100 ml IMI (Fig. 2A). However, honey bees exposed to THM exhibited a concentration-dependent increase in thiobarbituric (TBA) reactive substances (TBARS) (*JT* = 52.0; *p* ≤ 0.05; n = 40) (Fig. 2B). It should be noted that triglyceride levels (used to standardize TBARS values) remained unchanged in bees exposed to the various concentrations of IMI or THM (Table 2). The non-significant results on LPO following IMI exposure do not vouch for an unaffected redox state. For instance, Lopez-Antia *et al*. (2015)^30^, observed a higher superoxide dismutase activity in the red blood cells of *A. Rufa* although they did not observe LPO or a reduction of the glutathione redox system. As well, in a short term exposure study, Christen *et al*.^37^ recently showed that honey bees exposed to a highly IMI-contaminated syrup (up to 300 ng/ml) for 24 h to 72 h did not exhibit a clearly modified pattern in the expression of catalase; an enzyme implicated in the protection against free radical damages. Antioxidants can intervene before or after LPO depending on the sequence of oxidative events. Indeed, after IMI exposure, we noted that α-tocopherol levels showed a significant concentration-dependent diminution (*JT* = 23.0; *p* < 0.05; n = 37) (Table 2) as well as α-carotene, well known to be prone to oxygen singlet quenching^38^. Alternately, IMI could have induce the overexpression of various CYP450 implicated in the oxidation of many cellular constituents such as α-tocopherol and lipids^39^. Consequently, α-tocopherol could have been readily oxidized before any protective intervention on lipids. Still, we do not have a complete portrait of the modulations in honey bees’ antioxidant enzymes and their relative importance. The perturbed levels of dietary antioxidants by IMI and the increase in oxidative stress in bees by THM highlight the necessity of using a wider array of oxidative stress markers in future studies.

The retinoid metabolism (see Fig. 1) is finely regulated by oxidation-reduction processes and could be altered by oxidative damages, in turn leading to unbalanced retinoid compounds in the honey bee. Despite recent evidence of RA implications in the development of insects, few studies addressed the link between RA and the carotenoid-retinoid metabolism. So far, NinaB, an isomeroxygenase that combines carotene oxygenase and retinoid isomerase functions, was found in several insect species^40^ and appears to use many carotenoids such as α/β-carotene, β-cryptoxanthin, lutein and zeaxanthin as substrates in order to produce various proportions of RALD isomers^40,41^. In *D. melanogaster*, short chain dehydrogenases (SDRs) were shown to be biochemically similar to the human Rdh12^42^. These SDRs were assumed to convert all-*trans*-RALD or all-*trans*-3-hydroxy-RALD to ROL isomers and might also be implicated in the reverse reaction in some cell types^42^. Human Aldh1a orthologous genes found in some insects suggest potential enzyme candidates for the oxidation of RALD to RA^43^. In our study, no significant differences could be found for RALD between exposed and control groups of honey bees. These results could suggest that NinaB palliated the degradation of RALD due to its essential role in honey bees’ vision^44^. Yet, recent studies investigating the retinoid metabolism following agrochemicals exposure also reported an absence of a clear trend with RALD isomers (-*cis* and –*trans*) contents in honey bees^45,46^.

**Figure 3:**
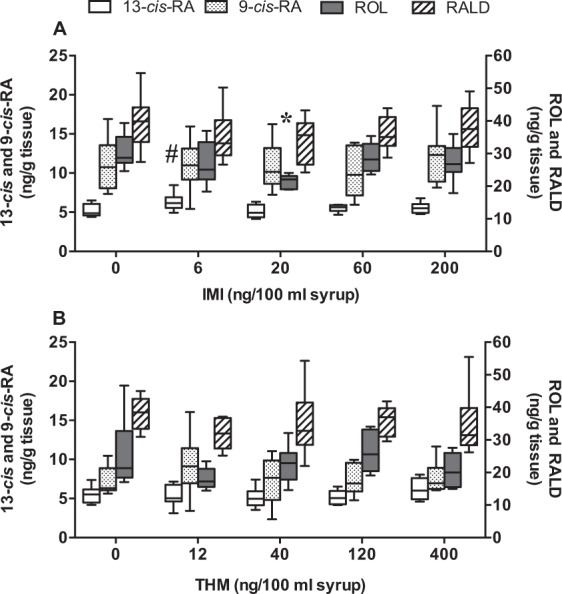
Retinoid contents (13/9-*cis*-RA, ROL and RALD) in honeybees exposed to increasing concentrations of IMI (**A**) and THM (**B**). Boxes (6–8 pools of 10 bees) extend from 25th to 75th percentile; line is the median and whiskers show the largest to smallest observed values. *p < 0.05 GLM one-way analysis of variance followed by Dunnett *t* test. ^#^*p* < 0.1 Kruskal–Wallis one-way analysis of variance followed by Dunn’s test.

Retinoid levels were previously shown to be modulated by various agrochemicals in amphibians, fishes, birds and mammals, and it seems that the honey bee retinoid system is not an exception, since it was affected by metolachlor, atrazine and glyphosate herbicides^47^. In our study, RALD contents were not changed by the exposure to IMI or THM (Fig. 3A and B) and a decrease of ROL contents was observed when bees were exposed to 20 ng/100 ml of IMI (*F*_4,29_ = 3.09; *p* < 0.05*)*. ROL was reported to neutralize oxidative damages to polyunsaturated fatty acids in membrane models of photoreceptor cells^48^. We believe that the antioxidant properties of ROL were sufficient to counteract a potential oxidative stress resulting from the exposure of honey bees to 20 ng/100 ml of IMI. At higher concentrations of IMI however, lipophilic compounds such as α-carotene, α-cryptoxanthin and α-tocopherol could have replaced ROL to neutralize oxidative damage protecting this important retinoid from decreasing. This hypothesis was supported by the significant dose dependent diminutions for α-carotene, α-cryptoxanthin and α-tocopherol (Table 2).

It should be noted that although the chromatography method we used allows for the detection of all-*trans*-RA, this compound was not detected in enough samples to be submitted to statistical tests. However, the statistical analysis reported a difference in 13-*cis*-RA levels (*KW* = 7.83, *p* < 0.1, n = 37). In fact, according to Dunn’s test, there was a tendency of increased levels for the IMI dose of 6 ng/100 ml (*p* < 0.1) but not higher doses (Fig. 3A). The increase of 13-*cis*-RA level should be considered with caution because of the statistical result. This non-linear effect could be related to RA isomerization and genetic reprogamming. In mammals, some authors have argued that the conversion of RA from –*trans* to –*cis* isomer prevented the overactivation of nuclear receptors RAR and RXR^49^. In invertebrates, very little is known about such isomerization. In this study, all-*trans* RA was not measured in enough samples to observe the ratio between all-*trans*-RA and 13-*cis*-RA which could have helped to better interpret the non-linear effect of IMI on 13-*cis*-RA. According to Pisa *et al*. (2014), NEOCs are among the chemicals that produce non-linear effects at (very) low doses associated with genetic reprogramming leading to unexpected responses^50^. Such non-linear effects at very low concentrations of IMI were reported on the survival of *Drosophila melanogaster*^51^ and one may think that genetic reprogramming could also be responsible for the honey bee 13-*cis*-RA fluctuating levels observed in our study.

**Figure 4:**
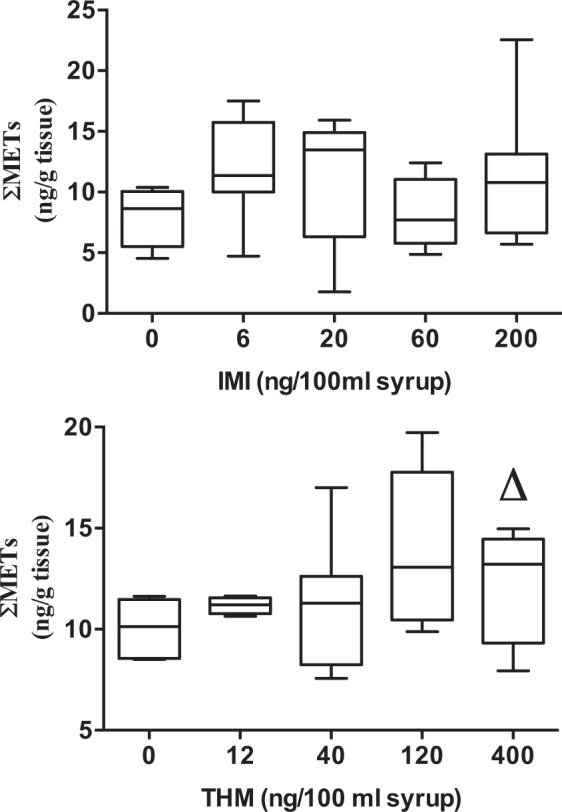
Retinoid contents (sum of 13-*cis*-4-oxo and all-*trans*-4-oxo-RA) in honeybees exposed to increasing concentrations of IMI (**A**) and THM (**B**). Boxes (5–8 pools of 10 bees, except 4 pools for 12 ng/100 ml) extend from 25th to 75th percentile; line is the median and whiskers show the largest to smallest observed values. ^Δ^*p* < 0.05 Jonkheere-Terpstra trend test.

More significant results were obtained with RA metabolites (ΣMETs: 13-*cis*-4-oxo-RA and all*-trans*-4-oxo-RA). In honey bees exposed to THM, a positive significant trend was found (*JT* = 35.0; *p* < 0.05; n = 31) (Fig. 4B) indicating increased levels of 13-*cis-*4-oxo-RA and all*-trans-*4-oxo-RA with increased THM doses. Such increases in 13-*cis*-4-oxo-RA have also been reported for bees exposed *in vitro* to a mixture of agrochemical contaminants (atrazine, glyphosate and cadmium)^45^ and for bees exposed to a variety of metals, herbicides and insecticides in maize growing areas^46^.

The reported decreased values of ROL by IMI and increased concentrations of TBARS, 13-*cis*-RA and ΣMETs by THM are in step with the two studies cited above and lead us to consider that NEOCs, like other agricultural contaminants, have a role to play in the RA oxidation pathway. From this perspective, the activities of CYP450 implicated in the formation of RA metabolites, Rdh, RALDH and Rrd could be further investigated.

Early studies demonstrated that honey bee sensitivity to pesticides may vary according to dietary resources^52^ and most probably life stages. For these reasons and to minimize variations that might have been introduced by geographical localizations of hives, special care was taken to capture bees from the same beehive and on frames without brood to ensure roughly similar ages of adult bees. Levels of carotenoids and retinoids reported here are similar to those published earlier^45,47^, but α-tocopherol levels were above those measured in 2013^12^. Variations in carotenoids, ROL and α-tocopherol levels were measured in many insects across seasons, developmental stages and ages (in days) and in some cases were postulated to be diet-related^53–55^. Taking into account the considerable shift in flowering crops between mid-July and the end of July in Quebec and the variation in pollen constituents (carotenoids and lipids) across plant species and seasons^15^, this likely introduced dietary variations between exposures to IMI (July 30 to August 9) and THM (July 17 to 27). In fact, different levels of triglycerides, vitamin E, carotenoids, LPO and ROL were observed between the control groups of honey bees exposed to IMI and THM (Table 2). These differences could originate from lower seasonal intakes of antioxidants and (pro)-vitamins, resulting in higher TBARS levels in IMI controls compared to those in the THM exposure. Such variations in physiological parameters were not surprising and should be considered while interpreting results. They further focus the importance of 1) characterizing exposure responses temporally (developmental stages and seasons) and 2) conducting broader-scale studies on multiple hives and through different honey bee species and sub-species before providing robust biomarkers in honey bees.

While many studies conducted on the honey bees exposed to NEOCs reported changes in immunity^56^ and behavior, such as mobility, memory/learning capacities and orientation (see review by Decourtye and Devillers 2010^57^), there is a consistent lack of biochemical investigations limiting the mechanistic understanding the toxicity of NEOCs. Their typical toxicity is mediated by their irreversible binding to nAChRs, which have also been hypothesized to cause oxidative stress^58^. In addition, a second mechanism of toxicity was proposed for IMI by its electron affinity and the release of NO_2_ leading to oxidative stress^59^. These two toxic pathways – the putative NAChRs-mediated toxicity of NEOCs and their suspected nitrosative effect – may lead to non-linear toxicity relationships. Also, some rather interesting insights suggest that memory and immunity disorders induced by NEOCs could be mediated by the retinoids as these compounds were recently linked to the establishment of long-term memory in snails^60^ and the maintenance of gut microflora in mice^61^. In fact, gut microflora has been proposed as a primary immune defense mechanism against microsporidium for the honey bee^62^. Our research focused on adult honey bees, yet the possibility of early damages occurring during larval and pupal stages merits investigation and should ultimately be addressed as they may affect the later stages. Developmental abnormalities and early cell death in optical lobes as well as alterations in cellular ultrastructure (midgut, Malpighian tubules and Kenyon cells) were documented in honey bee larvae following sub-lethal THM exposure^63,64^. Although retinoid metabolism and functions during insect larval and pupal stages are mostly unknown, retinoids have been shown to be crucial in the development of few insects^17–19^. Alteration of the retinoid metabolism and further RA signaling could be important in the reorganization of organs of holometabolous insects and could explain these previous developmental abnormalities. Both honey bee larvae and adults consume nectar and are exposed to various levels of NEOCs for prolonged period of time and at different stages of development. Thus, it is legitimate to think that NEOCs could affect several physiological and developmental parameters through different retinoid-mediated mechanisms in the honey bee. Future studies should aim to characterize the retinoid-carotenoid metabolism in insects and could help to understand the contaminant-induced oxidation processes in honey bees (adults and larvae) leading to unbalanced retinoid levels.

In this study, we raised concern about the NEOCs-mediated consequences on retinoid profiles at low and environmentally relevant concentrations in the honey bee. To our knowledge, our research is the first to report disorders in the retinoid-carotenoid metabolism following NEOCs exposure in *A. mellifera*. Our results showed that honeybees exposed for 10 days to environmentally relevant concentrations of technical grade neonicotinoids exhibited a concentration-dependent decrease of α-carotene and α-tocopherol, and an increase in TBARS and the sum of RA metabolites. Of note, these changes occurred at concentrations below those that affected AChE activity, thus demonstrating the sensitivity of the retinoid pathway. Therefore, we draw attention to the retinoids as novel, potential and sensitive biomarkers to assess honey bee health and possibly the health of other insects. Overall, we believe that temporal evolution of these changes, during and after the 10-day exposure period, as well as identification of the bees’ compartments or organs affected by these changes merit further investigation.

## Materials and Methods

### Chemicals

Standards for carotenoids (lutein, zeaxanthin, α-cryptoxanthin, β-cryptoxanthin, α-carotene and β-carotene) were purchased from DHI Labs (Hørsholm, Denmark). Acetylthiocholine iodide, butylated hydroxytoluene (BHT), cholesterol (≥99%), 5,5′-dithio-bis 2-nitrobenzoic acid (DTNB), malondialdehyde (MDA), retinoids (13-*cis*-RA, 9-*cis*-RA, all-*trans*-RA, all-*trans*-ROL and RALD), sodium phosphate monobasic, t-octylphenoxypolyethoxyethanol (Triton X-100), tris[hydroxymethyl]aminomethane (Trizma base), vitamin E (α-tocopherol), antipain dihydrochloride, pepstatin A, 2-thiobarbituric acid (TBA) and technical grade IMI and THM were purchased from Sigma-Aldrich Ltd. (Oakville, ON, Canada). Standards for 13-*cis-*4-oxo-RA and all-*trans* 4-oxo-RA were synthetized by a method already published^65^. Ascorbic acid, pyridine, bovine albumin serum, sodium deoxycholate, trichloroacetic acid and sodium dodecyl sulfate were procured from Fisher Scientific (Montreal, QC, Canada). HPLC-grade solvents were used.

### Honey bee exposure

Cage exposure was as described by Gauthier *et al*. (2015)^66^ with few modifications. To avoid differences between colonies (e.g., genetics, behavior, etc.), bees were selected from frames without brood (same beehive) during summer 2014 for exposure to THM (July 17 to 27) and IMI (July 30 to August 9). They were maintained in a temperature-controlled room (darkness, 25 °C ± 1, 55 ± 5% relative humidity) and four replicates (cages) per concentration were tested (30 bees per cage) for a total of 120 bees per concentration. Contaminated syrups were prepared by dissolving technical grade (that is without surfactants of commercial formulations) insecticides in 50% w/w sucrose solution and kept at −20 °C until use. Required volumes of contaminated syrups were thawed daily and aliquoted in feeders (1.5 ml). Feeders were weighted before and after changes (to monitor syrup consumption) for the 10-day exposure. Imidacloprid and THM were dissolved in nanopure water (0.2 mg/ml, stock solution) and diluted 1/400 before being added to the sugar syrup. Concentrations of contaminated syrups were tested by chemical analysis performed by the Laboratoire d’expertises et d’analyses alimentaires (Ministère de l’Agriculture, des Pêcheries et de l’Alimentation du Québec). Levels of exposures were based on previous work and did not exceed 3% of the LD_50_ (3.7–6.12 ng/bee)^12,67^. The interest of regulatory agencies in the LOAEC value of NEOCs and the reported behavioral and biochemical alterations at the lowest dose (0.08 ng active matter/bee) in the study published by Boily *et al*. (2013)^12^ was decisive in the selection of a much lower interval of concentrations for the present experiment. Syrup concentrations were as follows: 0, 6, 20, 60 and 200 ng/100 ml for IMI and 0, 12, 40, 120 and 400 ng/100 ml for THM. Daily insecticide intake per bee (pg/bee) was estimated by multiplying syrup concentration (pg/μl) per daily volume of syrup intake per bee (μl/bee). Control cages were supplied with sucrose solution only. At the end of the 10-day exposure, the bees were euthanized by placing the cages in an insulated container with dry ice for 5 min and conserved at −80 °C. Bees from each insecticide concentration were randomly mixed before biochemical analysis.

### Acetylcholinesterase (AChE) assay

#### Bee head extracts

A method was adapted from Boily *et al*. (2013)^12^ using four bee heads instead of eight to ten. The heads were cut, weighed and homogenized in 1.0 ml of low salt-triton (LST) buffer (10 mM NaCl, 1% w/v Triton X-100, 15 mM sodium phosphate, pH 7.3). Homogenates were centrifuged at 100,000 × *g* for 1 h at 4 °C. Pellets were rinsed once with 200 μl LST buffer, and rinses were combined with their supernatant. Pellets were resuspended with 500 μl of LST buffer. The extracts (combined supernatants and rinses; about 1.7 ml) were used for AChE assay and protein analysis.

#### Enzymatic assay

The AChE assay was done according to Boily *et al*. (2013)^12^ using 30 μl of head extracts. Analyses (n = 9–10) were performed for each concentration of insecticide and their respective control group. The absorbance read (410 nm) at 10 min was substracted from the absorbance read at 35 min to obtain AChE activity over 25 min. All readings were corrected by the absorbance of blank reaction tubes. Values of AChE activity were expressed as unit (U)/g protein (1 U = 1 mAbs/h). Readings were done with an Infinite M1000 Quadruple Monochromator Microplate Reader (Tecan Group Ltd., Durham, NC, USA).

#### Protein assay

Proteins were quantified with the Pierce BCA Protein Assay Kit (Fisher Scientific, Montreal, QC, Canada).

### Whole bee homogenates and extracts

Whole bee homogenates were prepared as described earlier with few modifications^66^. Briefly, 10 bees were weighed, rinsed with acetone (to remove pollen and other residues) and homogenized on ice in a 12-ml glass tube with 4 ml of a freshly made homogenization solution (phosphate buffer saline containing 0.05 mM antipain, 5 µM pepstatin A and 0.5% ascorbic acid, pH = 7.5). Two ml of the resulting homogenate were transferred into a second 12-ml glass tube and kept on ice for retinoids, carotenoids and α-tocopherol analysis by HPLC. The remaining homogenate was centrifuged at 4 °C, 9600 × *g* for 10 min. The supernatant (whole bee extract) was aliquoted and frozen at −80 °C for subsequent lipid peroxidation (LPO) and triglyceride quantifications. Eight pools of 10 bees were prepared for each concentration of insecticides tested.

Prior to HPLC analysis, whole bee homogenate (2 ml) was mixed with 1 ml of MeOH containing 0.1% BHT, vortexed for 30 s and extracted three times with a 50:50 hexane (containing 0.1% BHT)/acetone solution. A volume of 4 ml was used for the first extraction, while 3 ml were used for the second and the third extractions. After each addition of the hexane/acetone solution, the tube was vortexed for 90 s and centrifuged for 5 min at 1625 × *g*. In order to reduce the evaporation time, the supernatant (1.5 ml) of the first extraction was collected and distributed evenly between two 5-ml disposable glass tubes and evaporated to dryness for 10 min in a vacufuge (Eppendorf^TM^, Fisher Scientific, Ottawa, Canada) at 45 °C. This procedure was repeated with the second extraction (1.7 ml) and the third extraction (1.5 ml). The residue in each tube was resuspended in 100 μl acetonitrile and vortexed for 30 s. From each tube, 80 μl were collected and combined in a 5-ml disposable glass tube, vortexed for 10 s and injected in the HPLC system.

### HPLC analysis of α-tocopherol, carotenoids and retinoids

Two injections were performed simultaneously in two reversed-phase HPLC systems (Water Corporation, Milford, MA) connected to Empower Pro software (version 5.0), a model 510 pump and a model 7725i Rheodyne injector.

#### HPLC 1 – Carotenoids and α-tocopherol

Carotenoids (30 μl) were detected at 445 nm using the chromatographic conditions of Hedrei-Helmer *et al*. (2015)^47^. α-Tocopherol was detected at 292 nm using the same chromatographic method.

#### HPLC 2 – Retinoids

Retinoids (80 µl) were separated on an Ace C18 analytical column, 4.6 × 150 mm, 3 μm (Canadian Life Science, Peterborough, ON, Canada), using a recently published method^45^.

### Lipid peroxidation (LPO) quantification

Oxidative stress was evaluated using 200 µl of whole bee extracts and the TBARS method presented earlier^47^. This method relies on the quantification of MDA, a product of LPO that reacts with TBA. TBARS values were reported relative to triglyceride level to correct for any difference in lipids between samples, as suggested by Jentzsch *et al*. (1996)^68^. Triglycerides were measured with the EnzyChromTM Triglyceride Assay Kit from BioAssay Systems (Cat# ETGA-200) using 40 μl of the same extract.

### Statistical analyses

Survival was tested with Log-rank (Mantel-Cox) tests comparing curves from each concentration to their respective control group. Mean daily syrup consumption per bee, whole body mass, retinoids (13-*cis-*4-oxo-RA, all-*trans*-4-oxo-RA, 13-*cis*-RA 9-*cis*-RA, all*- trans*-RA, all*-trans-*ROL and RALD), carotenoids (lutein, zeaxanthin, α-/β-cryptoxanthin, α-/β-carotene), α-tocopherol, triglycerides, LPO (TBARs) and AChE activity values were compared using the GLM (general linear model) one-way analyses of variance. The normality of data distribution and the homogeneity of variance were addressed with Shapiro-Wilk and Levene tests respectively, and outliers were identified and discarded following a Grubb’s test (<2% of data for all parametric tests). In case of unequal variance, a Welch’s ANOVA was performed. Significant models (*p* < 0.05) were followed up on with a Dunnett *t* test against the control group. When transformations failed to normalize the distribution of data, non-parametric Kruskal-Wallis (KW) tests were used, followed by Dunn’s multiple comparisons test. The non-parametric Jonckheere-Terpstra (JT) test, based on the medians ordered in a particular direction, was performed to detect a possible trend related to a concentration-response relationship. Statistics were performed with SPSS 18.0 software (IBM Corporation, NY, USA).

## Electronic supplementary material

Dataset 1

## Data Availability

The datasets generated and analysed during the current study are available from the corresponding author on reasonable request.

## References

[1] Jeschke P, Nauen R, Schindler M, Elbert A (2011). Overview of the status and global strategy for neonicotinoids. J. Agric. Food Chem..

[2] Main Anson R., Headley John V., Peru Kerry M., Michel Nicole L., Cessna Allan J., Morrissey Christy A. (2014). Widespread Use and Frequent Detection of Neonicotinoid Insecticides in Wetlands of Canada's Prairie Pothole Region. PLoS ONE.

[3] Giroux, I. & Pelletier, L. *Bilan dans quatre cours d’eau de zones en culture de maïs et de soya en 2008, 2009 et 2010* (2012).

[4] Mullin Ca (2010). High levels of miticides and agrochemicals in North American apiaries: implications for honey bee health. PLoS One.

[5] Krupke CH, Hunt GJ, Eitzer BD, Andino G, Given K (2012). Multiple routes of pesticide exposure for honey bees living near agricultural fields. PLoS One.

[6] Schneider CW, Tautz J, Grünewald B, Fuchs S (2012). RFID Tracking of sublethal effects of two neonicotinoid insecticides on the foraging behavior of Apis mellifera. PLoS One.

[7] Han Peng, Niu Chang-Ying, Lei Chao-Liang, Cui Jin-Jie, Desneux Nicolas (2010). Use of an innovative T-tube maze assay and the proboscis extension response assay to assess sublethal effects of GM products and pesticides on learning capacity of the honey bee Apis mellifera L. Ecotoxicology.

[8] Alaux C (2010). Interactions between Nosema microspores and a neonicotinoid weaken honeybees (Apis mellifera). Environ. Microbiol..

[9] Williams GR (2015). Neonicotinoid pesticides severely affect honey bee queens. Sci. Rep..

[10] Wu JY, Anelli CM, Sheppard WS (2011). Sub-lethal effects of pesticide residues in brood comb on worker honey bee (Apis mellifera) development and longevity. PLoS One.

[11] Li Z (2017). Differential physiological effects of neonicotinoid insecticides on honey bees: A comparison between Apis mellifera and Apis cerana. Pestic. Biochem. Physiol..

[12] Boily M, Sarrasin B, Deblois C, Aras P, Chagnon M (2013). Acetylcholinesterase in honey bees (Apis mellifera) exposed to neonicotinoids, atrazine and glyphosate: laboratory and field experiments. Environ. Sci. Pollut. Res. Int..

[13] Badiou-Bénéteau A (2012). Development of biomarkers of exposure to xenobiotics in the honey bee Apis mellifera: application to the systemic insecticide thiamethoxam. Ecotoxicol. Environ. Saf..

[14] Felton GW, Summers CB (1995). Antioxidant systems in insects. Arch. Insect Biochem. Physiol..

[15] Mărgăoan R (2014). Predominant and secondary pollen botanical origins influence the carotenoid and fatty acid profile in fresh honeybee-collected pollen. J. Agric. Food Chem..

[16] Albalat R (2009). The retinoic acid machinery in invertebrates: ancestral elements and vertebrate innovations. Mol. Cell. Endocrinol..

[17] Nakamura A (2007). Effects of retinoids and juvenoids on moult and on phenoloxidase activity in the blood-sucking insect Rhodnius prolixus. Acta Trop..

[18] Halme A, Cheng M, Hariharan IK (2010). Retinoids regulate a developmental checkpoint for tissue regeneration in Drosophila. Curr. Biol..

[19] Němec V, Kodrik D, Matolin S, Lauferg H (1993). Juvenile hormone-like effects of retinoic acid in insect metamorphosis, embryogenesis and reproduction. J. Insect Physiol..

[20] Sukiban J, Bräunig P, Mey J, Bui-Göbbels K (2014). Retinoic acid as a survival factor in neuronal development of the grasshopper, Locusta migratoria. Cell Tissue Res..

[21] Boily, M., Marjolaine, B. & Spear, P. A. Rétinoïdes: Biomarqueurs et base moléculaire d’effets de substances toxiques. In *Écotoxicologie Moléculaire: Principes fondamentaux et perspectives de développements* 197–256 (Presses de l’Université du Québec, 2004).

[22] Blacquière T, Smagghe G, van Gestel CaM, Mommaerts V (2012). Neonicotinoids in bees: a review on concentrations, side-effects and risk assessment. Ecotoxicology.

[23] OECD. *Proposal for a new guidelines for the testing of chemicals; Honeybee (Apis mellifera L.), chronic oral toxicity test 10* *day feeding test in the laboratory* (2016).

[24] Morakchi S, Maïza A, Farine JP, Aribi N, Soltani N (2005). Effect of a neonicotinoid insecticide (acetamiprid) on acetylcholinesterase activity and cuticular hydrocarbons profil in german cockroaches. Commun. Agric. Biol. Sci..

[25] Azevedo-Pereira HMVS, Lemos MFL, Soares AMVM (2011). Effects of imidacloprid exposure on Chironomus riparius Meigen larvae: Linking acetylcholinesterase activity to behaviour. Ecotoxicol. Environ. Saf..

[26] Mesnage Robin, Defarge Nicolas, Spiroux de Vendômois Joël, Séralini Gilles-Eric (2014). Major Pesticides Are More Toxic to Human Cells Than Their Declared Active Principles. BioMed Research International.

[27] Puglis HJ, Boone MD (2011). Effects of technical-grade active ingredient vs. commercial formulation of seven pesticides in the presence or absence of UV radiation on survival of green frog tadpoles. Arch. Environ. Contam. Toxicol..

[28] Jemec A (2007). Comparative toxicity of imidacloprid, of its commercial liquid formulation and of diazinon to a non-target arthropod, the microcrustacean Daphnia magna. Chemosphere.

[29] Milani A, Basirnejad M, Shahbazi S, Bolhassani A (2017). Carotenoids: biochemistry, pharmacology and treatment. Br. J. Pharmacol..

[30] Lopez-Antia A, Ortiz-Santaliestra ME, Mougeot F, Mateo R (2015). Imidacloprid-treated seed ingestion has lethal effect on adult partridges and reduces both breeding investment and offspring immunity. Environ. Res..

[31] Kiefer, C., Sumser, E., Wernet, M. F. & Lintig, J. Von. A class B scavenger receptor mediates the cellular uptake of carotenoids in Drosophila. **99** (2002). 10.1073/pnas.162182899 PMC124981 12136129

[32] Ford, K. A., Gulevich, A. G., Swenson, T. L. & Casida, J. E. Neonicotinoid Insecticides: Oxidative Stress in Planta and Metallo-oxidase Inhibition. 4860–4867 (2011). 10.1021/jf200485k 21476569

[33] Malev O, Klobučar RS, Fabbretti E, Trebše P (2012). Comparative toxicity of imidacloprid and its transformation product 6-chloronicotinic acid to non-target aquatic organisms: Microalgae Desmodesmus subspicatus and amphipod Gammarus fossarum. Pestic. Biochem. Physiol..

[34] Ge W (2015). Oxidative stress and DNA damage induced by imidacloprid in zebrafish (Danio rerio). J. Agric. Food Chem..

[35] Kapoor U, Srivastava MK, Srivastava LP (2011). Toxicological impact of technical imidacloprid on ovarian morphology, hormones and antioxidant enzymes in female rats. Food Chem. Toxicol..

[36] Bal R (2012). Assessment of imidacloprid toxicity on reproductive organ system of adult male rats. J. Environ. Sci. Health. B..

[37] Christen Verena, Mittner Fabian, Fent Karl (2016). Molecular Effects of Neonicotinoids in Honey Bees (Apis mellifera). Environmental Science & Technology.

[38] Di Mascio P, Murphy ME, Sies H (1991). Antioxidant defense systems: the role of carotenoids, tocopherols and thiols. Am. J. Clin. Nutr..

[39] Derecka Kamila, Blythe Martin J., Malla Sunir, Genereux Diane P., Guffanti Alessandro, Pavan Paolo, Moles Anna, Snart Charles, Ryder Thomas, Ortori Catharine A., Barrett David A., Schuster Eugene, Stöger Reinhard (2013). Transient Exposure to Low Levels of Insecticide Affects Metabolic Networks of Honeybee Larvae. PLoS ONE.

[40] Oberhauser V, Voolstra O, Bangert A, von Lintig J, Vogt K (2008). NinaB combines carotenoid oxygenase and retinoid isomerase activity in a single polypeptide. Proc. Natl. Acad. Sci. USA.

[41] Babino D (2016). The biochemical basis of vitamin A3 production in arthropod vision. ACS Chem. Biol..

[42] Belyaeva OV, Lee S, Kolupaev OV, Kedishvili NY (2009). Identification and characterization of retinoid-active short-chain dehydrogenases/reductases in Drosophila melanogaster. Biochim. Biophys. Acta.

[43] Albalat R, Cañestro C (2009). Identification of Aldh1a, Cyp26 and RAR orthologs in protostomes pushes back the retinoic acid genetic machinery in evolutionary time to the bilaterian ancestor. Chem. Biol. Interact..

[44] Goldsmith TH (2013). Evolutionary tinkering with visual photoreception. Vis. Neurosci..

[45] Jumarie C, Aras P, Boily M (2017). Mixtures of herbicides and metals affect the redox system of honey bees. Chemosphere.

[46] Boily M, Aras P, Jumarie C (2017). Foraging in maize field areas: A risky business?. Sci. Total Environ..

[47] Hedrei-Helmer S, Kerbaol A, Aras P, Jumarie C, Boily M (2015). Effects of realistic doses of atrazine, metolachlor, and glyphosate on lipid peroxidation and diet-derived antioxidants in caged honey bees (Apis mellifera). Environ. Sci. Pollut. Res. Int..

[48] Keys SA, Zimmerman WF (1999). Antioxidant activity of retinol, glutathione, and taurine in bovine photoreceptor cell membranes. Exp. Eye Res..

[49] Armstrong JL, Redfern CPF, Veal GJ (2005). 13-cis Retinoic acid and isomerisation in paediatric oncology - Is changing shape the key to success?. Biochem. Pharmacol..

[50] Pisa LW (2014). Effects of neonicotinoids and fipronil on non-target invertebrates. Environ. Sci. Pollut. Res..

[51] Charpentier G (2014). Lethal and sublethal effects of imidacloprid, after chronic exposure, on the insect model drosophila melanogaster. Environ. Sci. Technol..

[52] Wahl O, Ulm K (1983). Influence of pollen feeding and physiological condition on pesticide sensitivity of the honey bee Apis mellifera carnica. Oecologia.

[53] Karpunina NN (1961). Changes in carotenoid content in the body of Colorado potato beetle during imaginal life. Dokl. Akad. Nauk U.S.S.R.

[54] Giovannucci, D. R. & Stephenson, R. S. Identification and distribution of dietary precursors of the Drosophila visual pigment chromophore: analysis of carotenoids in wild type and ninaD mutants by HPLC. **39**, 219–229 (1999). 10.1016/s0042-6989(98)00184-9 10326132

[55] Kara T (2013). Seasonal variation of vitamin and sterol content in chironomidae larvae. Pakistan J. Biol. Sci..

[56] Brandt A, Gorenflo A, Siede R, Meixner M, Büchler R (2016). The neonicotinoids thiacloprid, imidacloprid, and clothianidin affect the immunocompetence of honey bees (Apis mellifera L.). J. Insect Physiol..

[57] Decourtye A, Devillers J (2010). Ecotoxicity of neonicotinoid insecticides to bees. in. Advances in experimental medicine and biology.

[58] Farooqui T (2013). A potential link among biogenic amines-based pesticides, learning and memory, and colony collapse disorder: A unique hypothesis. Neurochem. Int..

[59] Kovacic P, Somanathan R, Nguyen H, Lopez AC (2017). Unifying mechanism involving neonicotinoids for bee toxicity: electron affinity, nitrogen dioxide, oxidative stress, reactive oxygen species. Adv. Biochem. Biotechnol..

[60] Rothwell CM, Simmons J, Peters G, Spencer GE (2014). Neurobiology of learning and memory novel interactive effects of darkness and retinoid signaling in the ability to form long-term memory following aversive operant conditioning. Neurobiol. Learn. Mem..

[61] Pantazi E, Marks E, Stolarczyk E, Lycke N, Noelle RJ (2016). Retinoic acid signaling in B-cells is essential for oral immunization and microflora composition. J. Immunol..

[62] Dussaubat C (2012). Gut pathology and responses to the microsporidium Nosema ceranae in the honey bee Apis mellifera. PLoS One.

[63] Friol PS, Catae AF, Tavares DA, Malaspina O, Roat TC (2017). Can the exposure of Apis mellifera (Hymenoptera, Apiadae) larvae to a field concentration of thiamethoxam affect newly emerged bees?. Chemosphere.

[64] Tavares DA (2015). *In vitro* effects of thiamethoxam on larvae of Africanized honey bee Apis mellifera (Hymenoptera: Apidae). Chemosphere.

[65] McKenzie R, Hellwege D, McGregor M, Nelson E (1979). Oxidation and isomerisation of retinoic acid by iodine and light: A novel preparation of all-trans and 13-cis-4-oxo retinoic acid. Lipids.

[66] Gauthier M, Aras P, Jumarie C, Boily M (2016). Low dietary levels of Al, Pb and Cd may affect the non-enzymatic antioxidant capacity in caged honey bees (Apis mellifera). Chemosphere.

[67] Grimm, M., Sedy, K., Süßenbacher, E. & Riss, A. *Existing scientifi evidence of the effects of neonicotinoid pesticides on bees* (2012).

[68] Jentzsch AM, Bachmann H, Fürst P, Biesalski HK (1996). Improved analysis on malondialdehyde in human body fluids. Free Radic. Biol. Med..

