# Supplementary material for: Chronic exposure to imidacloprid or thiamethoxam neonicotinoid causes oxidative damages and alters carotenoid-retinoid levels in caged honey bees (Apis mellifera)
Source: Sci Rep. 2018 Nov 2;8:16274. doi: 10.1038/s41598-018-34625-y (PMC6214897; doi:10.1038/s41598-018-34625-y)
Supplement: Supplementary file 1 — Dataset 1 [file 41598_2018_34625_MOESM1_ESM.pdf]

Chronic exposure to imidacloprid or thiamethoxam neonicotinoid causes oxidative damages and alters carotenoid-retinoid levels in caged honey bees (*Apis mellifera*)

Maxime Gauthier[1], Aras Philippe[1], Paquin Joanne[2] and Monique Boily[1*]

[1] Département des Sciences Biologiques, Université du Québec à Montréal, C.P. 8888, Succursale Centre-Ville, Montréal, Québec, Canada H3C 3P8

[2] Département de Chimie, Université du Québec à Montréal, C.P. 8888, Succursale Centre-Ville, Québec, Canada H3C 3P8

Corresponding author: Dr. Monique Boily, PhD

Département des Sciences Biologiques

Université du Québec à Montréal

C.P. 8888, Succursale Centre-Ville

Montréal, Québec, Canada H3C 3P8

Phone: 514-987-3000 (ext. 5605)

Fax: 514-987-4647

Email: boily.monique@uqam.ca

General informations

Exposure	Capture	Day 1	Sugar syrup volumic mass
**THM**	July 16th 2014	July 17th 2014	1.2251g/ml
**IMI**	July 30th 2014	Juy 31st 2014	1.2251g/ml

Additionnal information: Feeders were replaced at 17:00 everyday and humidity, temperature and mortality were recorded.

Mass (g) of syrup consumed by honeybees. Number on the second line are the dates

	**THM**	17	18	18	19	19	20	20	21	21	22	22	23	23	24	24	25	25	26	26	27
cage	ng/ 100ml	in	out	in	out	in	out	in	out	in	out	in	out	in	out	in	out	in	out	in	out
1	CTRL	2.6	1.8	2.8	2.1	2.5	1.6	2.4	1.8	2.5	1.6	2.8	1.8	2.8	1.9	2.7	2.1	2.5	1.6	2.5	1.5
1	CTRL	2.2	1.8	2.2	1.5	2.5	2.0	2.5	1.8	2.7	2.1	2.7	2.3	2.6	2.0	2.7	2.1	2.4	1.7	2.5	1.6
2	CTRL	2.1	1.2	2.6	2.1	2.6	1.6	2.5	1.8	2.6	1.9	2.7	2.0	2.8	1.8	2.6	1.6	2.5	1.8	2.7	1.9
2	CTRL	2.5	1.9	2.4	1.9	2.9	2.2	2.6	2.0	2.5	1.9	2.7	2.0	2.5	1.7	2.6	1.8	2.8	1.7	2.6	1.5
3	CTRL	2.7	2.0	2.2	1.6	2.5	1.9	2.7	2.0	2.7	2.1	2.7	2.0	2.6	1.8	2.8	2.3	2.7	2.2	2.5	1.3
3	CTRL	2.4	1.9	2.7	2.2	2.5	2.2	2.6	2.0	2.5	1.9	2.6	2.2	2.5	1.7	2.7	1.9	2.7	1.5	2.4	1.2
4	CTRL	2.5	1.6	2.6	1.9	2.5	1.8	2.3	1.6	2.5	1.7	2.6	2.0	2.6	2.1	2.7	2.1	2.5	1.6	2.9	1.9
4	CTRL	2.9	2.5	2.5	2.0	2.4	1.9	2.8	2.3	2.3	1.7	2.7	2.1	2.4	1.6	2.5	2.1	2.7	2.3	2.9	2.2
5	12	2.5	2.0	2.6	2.0	2.4	1.6	2.7	2.0	2.6	2.3	2.5	1.7	2.5	1.7	2.5	2.1	2.6	1.9	2.7	2.3
5	12	2.7	2.1	2.6	1.8	2.6	1.9	2.6	2.3	2.6	1.8	2.8	2.2	2.8	2.3	2.8	2.2	2.8	1.8	2.8	1.6
6	12	2.8	1.8	2.6	2.3	2.7	2.0	2.3	1.8	2.6	1.8	2.5	2.1	2.5	1.8	2.8	1.8	2.7	1.8	2.8	1.9
6	12	2.6	2.4	2.5	1.9	2.7	2.2	2.7	2.2	2.6	2.2	2.5	1.8	2.8	2.2	2.8	2.2	2.6	1.9	2.5	1.7
7	12	2.5	2.0	2.6	2.3	2.6	2.2	2.2	1.6	2.4	2.0	2.8	1.9	2.5	1.6	2.7	2.0	2.8	1.8	2.8	0.9
7	12	2.6	1.7	2.8	2.0	2.7	1.8	2.5	2.0	2.6	2.3	2.8	2.3	2.7	2.0	2.7	1.6	2.7	1.4	2.4	1.7
8	12	2.9	2.2	2.4	1.9	2.6	1.7	2.7	1.9	2.7	1.8	2.6	1.8	2.5	2.0	2.6	2.4	2.7	1.7	2.7	1.4
8	12	2.6	2.0	2.7	2.0	2.6	1.8	2.7	2.0	2.5	1.9	2.7	1.2	2.6	1.8	2.3	1.3	2.7	1.9	2.8	2.4
9	12	2.6	1.8	2.4	2.0	2.6	1.9	2.5	2.1	2.4	2.1	2.6	2.2	2.4	1.8	2.9	2.6	2.5	1.7	2.7	2.1
9	40	2.6	2.1	2.8	2.0	2.6	1.9	2.3	1.8	2.8	2.3	2.7	2.2	2.7	2.2	2.7	1.8	2.6	2.0	2.5	1.9
10	40	2.6	1.7	2.7	2.2	2.4	1.7	2.6	1.8	2.5	2.0	2.7	1.9	2.8	2.1	2.9	2.5	2.7	1.6	2.5	1.4
10	40	2.6	2.0	2.3	1.4	2.4	1.9	2.7	2.1	2.6	1.7	2.8	1.9	2.6	1.2	2.5	1.4	2.6	1.9	2.7	2.1
11	40	2.6	1.7	2.6	2.1	2.8	2.1	2.7	2.1	2.8	2.1	2.6	1.3	2.7	2.0	2.6	1.8	2.6	1.2	2.8	1.2
11	40	2.8	2.1	2.3	1.5	2.9	2.3	2.7	2.1	2.6	1.8	2.6	1.6	2.7	1.2	2.7	1.8	2.7	2.2	2.7	2.3
12	40	2.5	1.8	2.5	2.0	2.4	1.8	2.6	2.1	2.4	1.9	2.4	1.6	2.2	1.7	2.5	1.7	2.6	2.0	2.7	2.4
12	40	2.5	1.7	2.6	2.0	2.7	2.1	2.8	2.6	2.8	2.3	2.3	1.9	2.7	2.1	2.7	2.3	2.6	2.0	2.6	1.4
13	120	2.4	1.7	2.7	2.0	2.8	2.4	2.5	2.0	2.5	2.0	2.7	2.0	2.9	2.5	2.4	2.0	2.3	1.4	2.6	2.0
13	120	2.7	2.0	2.3	1.7	2.5	1.7	2.8	2.3	2.7	2.4	2.3	2.0	2.6	1.9	2.7	2.1	2.3	1.7	2.7	2.1
14	120	2.6	1.9	2.5	1.9	2.8	2.4	3.0	2.8	2.6	2.1	2.8	2.3	2.4	1.8	2.7	2.4	2.9	2.0	2.7	2.3
14	120	2.7	2.3	2.3	1.9	2.7	2.1	2.7	1.9	2.8	2.4	2.4	1.9	2.9	2.1	2.7	1.8	2.7	1.8	2.7	1.2
15	120	2.8	1.9	2.7	1.8	2.5	1.8	2.7	2.0	2.5	1.8	2.6	2.2	2.8	1.8	2.7	1.8	2.7	2.3	2.9	2.2
15	120	2.4	1.9	2.8	2.3	2.6	1.9	2.5	1.9	2.5	2.1	2.5	1.5	2.5	2.0	2.8	2.1	2.7	1.4	2.6	1.8
16	120	2.5	1.7	2.4	1.8	2.4	1.8	2.3	1.5	2.7	1.5	2.9	2.4	2.5	1.8	2.7	2.0	2.4	1.9	2.5	0.9
16	120	2.2	1.7	2.4	1.8	2.5	1.8	2.6	2.0	2.7	2.0	2.6	1.1	2.4	1.2	2.6	1.4	2.5	1.4	2.8	1.9
17	400	2.8	2.2	2.7	2.1	2.3	1.8	2.6	2.0	2.2	1.4	2.6	1.7	2.4	1.7	2.6	2.1	2.8	1.5	2.6	1.4
17	400	2.6	2.0	3.0	2.6	2.6	2.1	2.5	1.9	2.5	1.8	2.9	2.2	2.9	2.3	2.8	2.3	2.8	2.5	2.5	1.6
18	400	2.7	2.1	2.8	2.5	2.7	2.2	2.7	2.3	2.5	2.1	2.7	2.0	2.7	1.9	2.8	2.0	2.6	2.1	2.5	1.7
18	400	2.5	2.1	2.5	1.9	2.7	2.2	2.9	2.3	2.7	2.4	2.7	2.2	2.9	2.5	2.8	2.3	2.5	1.8	2.8	2.3
19	400	2.6	1.7	2.8	2.3	2.6	2.2	2.6	2.1	2.6	1.9	2.4	1.7	2.5	1.6	2.7	1.9	2.7	1.6	2.7	2.2
19	400	2.5	1.9	2.6	1.7	2.7	2.2	2.6	1.9	2.8	2.3	2.6	1.8	2.5	2.0	2.6	1.8	2.8	2.3	2.8	1.7
20	400	2.5	1.8	2.6	2.0	2.6	2.1	2.6	2.1	2.6	2.0	2.5	1.5	2.4	1.7	2.5	1.6	2.5	1.8	2.4	1.8
20	400	2.4	1.7	2.6	2.0	2.6	2.0	2.6	2.0	2.8	2.5	2.6	2.0	2.8	1.8	2.6	1.9	2.7	1.7	2.4	1.2
T^{o}		24.9		24.9		24.7		25.2		25.6		26.0		25.7		25.7		25.8		25.5	25.3
H.R		54		56		58		56		58		57		55		49		55		55	60

Cage	**THM** ng/100 ml	18	19	20	21	22	23	24	25	26	27	Total Mortality	N (end) 2010-07-26	N (beginning) 2010-07-16
1	CTRL											0	35	35
2	CTRL											0	35	35
3	CTRL											0	38	38
4	CTRL						1					1	34	35
5	12											0	38	38
6	12											0	37	37
7	12											0	35	35
8	12						2					2	33	35
9	40		1									1	34	35
10	40						1	1				2	35	37
11	40									1		1	33	34
12	40											0	36	36
13	120			1			1			1		3	33	36
14	120									1		1	34	35
15	120							2				2	33	35
16	120						1					1	37	38
17	400											0	38	38
18	400											0	35	35
19	400											0	35	35
20	400											0	36	36
	Total	0	1	1	0	0	6	3	0	3	0	14		

THM (ng/100 ml)	Replicate -	Protein (mg/g tissue)	AChE activity (mDO/g prot/h)
CTRL	1	29.8	0.747
CTRL	2	36.8	0.580
CTRL	3	48.4	0.337
CTRL	4	29.7	0.699
CTRL	5	28.6	0.502
CTRL	6	41.4	0.357
CTRL	7	48.7	0.378
CTRL	8	47.7	0.247
CTRL	9	28.1	0.290
CTRL	10	38.6	0.523
12	1	46.8	0.255
12	2	33.2	0.587
12	3	27.7	0.402
12	4	40.3	0.474
12	5	28.5	0.433
12	6	58.5	0.189
12	7	41.7	0.572
12	8	32.8	0.360
12	9	50.7	0.267
12	10	39.0	0.383
40	1	25.5	0.803
40	2	30.0	0.280
40	3	44.6	0.252
40	4	38.7	0.310
40	5	34.7	0.345
40	6	41.5	0.363
40	7	34.1	0.592
40	8	47.2	0.422
40	9	45.8	0.164
40	10	45.0	0.308
120	1	30.0	0.447
120	2	34.0	0.756
120	3	31.1	0.357
120	4	34.0	0.312
120	5	30.7	0.345
120	6	40.0	0.504
120	7	43.5	0.426
120	8	29.2	0.674
120	9	47.4	0.298
120	10	42.3	0.322
400	1	12.6	0.759
400	2	28.7	0.457
400	3	47.1	0.132
400	4	30.6	0.395
400	5	28.7	0.276
400	6	56.5	0.392
400	7	39.4	0.248
400	8	40.2	0.449
400	9	31.8	0.573
400	10	33.6	0.495

THM (ng/100ml)	Rep. -	Mass (g)	METs (ng/g. tiss.)	13-cis-RA (ng/g. tiss.)	9-cis-RA (ng/g. tiss.)	ROL (ng/g. tiss.)	RALD (ng/g. tiss.)
CTRL	1	1.354	11.63	6.32	8.88	36.06	ND
CTRL	2	1.402	10.43	5.70	6.05	20.14	33.38
CTRL	3	1.465	8.51	5.36	6.23	22.87	44.98
CTRL	4	1.405	ND	4.49	5.63	17.05	38.44
CTRL	5	1.570	8.57	5.65	10.46	17.87	30.89
CTRL	6	1.405	9.82	4.47	7.03	21.84	39.72
CTRL	7	1.433	11.41	7.38	ND	20.81	42.59
CTRL	8	1.386	ND	4.18	6.34	46.67	36.64
12	1	1.489	ND	4.93	7.33	17.46	27.69
12	2	1.420	19.61	7.17	16.05	17.04	25.15
12	3	1.497	ND	3.12	3.42	15.18	27.26
12	4	1.432	ND	4.65	10.43	18.74	32.84
12	5	1.493	11.65	5.13	6.95	14.38	31.11
12	6	1.440	11.13	7.05	9.11	16.65	37.11
12	7	1.455	10.65	5.90	11.45	23.45	36.92
12	8	1.380	11.29	4.62	ND	21.89	36.00
40	1	1.438	17.01	5.96	11.07	24.48	31.92
40	2	1.394	12.36	3.55	6.65	17.25	ND
40	3	1.547	8.24	5.77	7.71	19.41	21.95
40	4	1.325	ND	4.78	7.60	21.30	41.47
40	5	1.286	7.57	4.29	4.21	25.34	54.29
40	6	1.477	10.97	5.16	10.30	32.11	38.45
40	7	1.441	11.29	7.43	8.62	14.61	32.80
40	8	1.317	12.62	4.10	2.34	26.21	28.46
120	1	1.427	9.88	ND	ND	22.96	36.63
120	2	1.414	14.03	4.16	4.77	20.38	32.86
120	3	1.308	12.12	5.36	5.89	25.60	29.55
120	4	1.382	18.52	4.71	9.58	34.05	41.84
120	5	1.363	11.76	6.53	9.97	19.09	39.75
120	6	1.439	19.73	5.05	6.93	33.22	39.69
120	7	1.504	10.02	4.27	6.36	ND	30.34
120	8	1.462	15.49	5.95	6.93	32.81	37.32
400	1	1.377	14.29	5.74	6.92	22.24	32.58
400	2	1.326	7.94	4.60	6.95	15.08	29.70
400	3	1.438	ND	6.00	11.66	24.25	27.90
400	4	1.461	13.54	6.56	6.07	17.71	30.34
400	5	1.341	12.89	6.00	6.23	26.57	55.47
400	6	1.473	9.77	8.05	ND	16.88	40.19
400	7	1.356	14.97	4.63	8.93	14.94	26.17
400	8	1.391	ND	7.97	7.91	27.54	38.64

ND = Non detectable

THM	Rep.	lutein	zeaxanthin	A-crypto.	B-crypto.	A-carot.	B-carotene
(ng/100ml)	-	(ng/g. tiss.)	(ng/g. tiss.)	(ng/g. tiss.)	(ng/g. tiss.)	(ng/g. tiss.)	(ng/g. tiss.)
CTRL	1	251.82	113.17	87.05	43.55	231.72	238.13
CTRL	2	150.83	97.92	65.96	42.45	182.13	89.34
CTRL	3	211.07	74.45	84.71	39.39	130.09	198.12
CTRL	4	250.33	113.29	52.08	29.66	87.79	119.92
CTRL	5	150.09	54.87	50.74	32.34	104.98	243.33
CTRL	6	195.69	95.05	107.64	47.16	180.22	156.15
CTRL	7	189.82	80.08	81.26	46.74	171.73	243.56
CTRL	8	251.01	121.60	90.67	48.92	208.87	305.94
12	1	ND	118.65	72.39	60.71	120.03	120.03
12	2	115.97	91.61	57.87	32.90	137.25	48.21
12	3	183.19	75.69	57.90	44.93	145.82	131.75
12	4	191.07	94.58	72.42	38.08	142.33	130.06
12	5	130.47	59.32	48.41	29.69	137.63	189.51
12	6	180.59	103.42	80.53	46.85	195.14	148.32
12	7	158.80	77.04	51.59	36.61	270.06	186.06
12	8	146.19	87.10	70.23	42.63	184.78	103.13
40	1	133.50	66.78	59.76	35.05	127.25	158.19
40	2	203.28	97.96	74.45	43.53	193.94	267.43
40	3	142.72	61.59	64.99	37.24	112.34	201.85
40	4	154.41	87.97	84.27	45.77	148.40	164.70
40	5	170.03	66.02	87.15	35.39	159.56	196.17
40	6	112.73	44.31	65.64	31.51	140.83	152.78
40	7	243.58	98.15	90.89	41.06	125.22	282.48
40	8	179.86	63.39	61.45	36.12	109.43	200.44
120	1	258.87	98.79	62.03	47.34	228.42	355.66
120	2	156.30	59.32	43.99	29.42	100.87	222.08
120	3	142.69	75.55	83.74	38.98	143.78	198.29
120	4	244.54	106.28	95.66	51.26	207.61	395.89
120	5	178.46	77.82	89.76	36.24	179.89	184.37
120	6	213.92	101.57	75.03	42.67	177.97	214.15
120	7	159.38	86.74	65.89	40.48	154.20	193.19
120	8	202.15	96.90	61.65	44.95	160.51	167.92
400	1	192.93	88.00	70.07	44.06	221.60	206.81
400	2	168.67	89.51	72.50	44.52	176.89	212.94
400	3	182.43	77.08	ND	39.42	143.27	129.61
400	4	188.26	73.81	83.00	39.34	140.95	144.02
400	5	130.58	67.87	62.58	39.95	240.82	139.13
400	6	220.48	68.21	67.24	35.44	ND	ND
400	7	193.26	99.59	75.74	46.97	160.04	157.12
400	8	114.09	60.54	82.17	39.05	178.37	136.01

ND = Non detectable

Concentration of THM (ng/100ml)	Rep. -	A-tocopherol (ng/g. tissue)	Triglycerides (ng/g tissue)	TBARs (ng/ug of trigly)
CTRL	1	175.55	849.9	0.166
CTRL	2	136.41	801.3	0.168
CTRL	3	120.62	711.3	0.386
CTRL	4	104.45	641.7	0.304
CTRL	5	91.79	689.0	0.498
CTRL	6	104.27	649.4	0.270
CTRL	7	123.12	735.5	0.160
CTRL	8	174.02	636.8	0.324
12	1	148.61	629.3	0.326
12	2	107.82	755.4	0.509
12	3	106.45	687.4	0.255
12	4	151.58	625.8	0.453
12	5	115.16	528.7	0.670
12	6	156.01	750.9	0.355
12	7	139.30	691.3	0.181
12	8	149.29	788.1	0.364
40	1	108.20	771.2	0.578
40	2	187.09	624.5	0.298
40	3	113.98	611.3	0.417
40	4	160.51	599.4	0.501
40	5	116.16	835.8	0.340
40	6	80.86	709.2	0.111
40	7	195.50	645.4	0.323
40	8	95.73	743.4	0.257
120	1	186.28	718.7	0.162
120	2	121.44	734.5	0.176
120	3	138.87	924.5	0.417
120	4	218.18	598.3	0.196
120	5	143.69	673.7	0.356
120	6	169.50	614.5	0.510
120	7	108.22	490.5	0.736
120	8	137.37	634.0	0.316
400	1	143.10	698.3	0.437
400	2	148.50	550.8	0.669
400	3	162.75	557.6	0.592
400	4	140.91	555.8	0.605
400	5	134.47	606.4	0.367
400	6	145.00	756.3	0.487
400	7	129.02	695.5	0.347
400	8	ND	870.3	0.254

ND = Non detectable

Mass (g) of syrup consumed by honeybees. Number on the second line are the dates

	**IMI**	30	31	31	1	1	2	2	3	3	4	4	5	5	6	6	7	7	8	8	9
cage	ng / 100ml	in	out	in	out	in	out	in	out	in	out	in	out	in	out	in	out	in	out	in	out
1	60	2.7	2.1	2.5	2.1	2.8	2.3	2.9	2.1	2.6	1.6	2.7	1.8	2.3	1.4	2.5	1.8	2.5	1.6	2.6	1.8
1	60	2.5	2.1	2.5	1.8	2.7	1.8	2.6	1.9	2.9	2.1	2.8	2.1	2.7	1.7	2.8	1.7	2.8	2.1	2.6	1.8
2	60	2.6	1.9	2.3	1.9	2.4	1.5	2.4	1.4	2.9	2.5	2.5	1.6	2.3	1.4	2.6	1.5	2.7	1.6	2.7	1.7
2	60	2.9	2.4	2.4	1.4	2.6	2.1	2.6	2.1	2.7	1.5	2.3	1.6	2.6	1.8	2.8	2.2	3.0	2.1	2.8	1.6
3	60	2.6	2.0	2.5	1.8	2.7	1.9	2.5	1.7	2.8	2.0	2.8	2.0	2.4	1.4	2.6	1.9	2.7	2.2	2.8	1.7
3	60	2.6	1.9	2.6	1.7	2.3	1.8	2.8	2.3	2.6	1.8	2.5	1.3	2.8	1.5	2.8	1.7	2.7	1.4	2.7	1.8
4	60	2.3	1.8	2.5	1.7	2.7	2.0	2.7	1.7	2.7	1.7	2.7	1.8	2.6	1.5	2.5	1.8	2.7	1.9	2.8	2.2
4	60	2.6	2.0	2.5	1.7	2.8	2.1	2.5	1.6	2.2	1.0	2.7	1.8	2.5	1.7	2.8	2.0	2.8	2.3	2.4	1.6
5	200	2.5	1.9	2.8	2.3	2.7	2.3	2.8	2.0	2.7	2.1	2.6	2.0	2.6	1.8	2.8	1.5	2.6	1.5	2.7	1.4
5	200	2.8	2.4	2.8	2.0	2.5	2.0	2.6	2.2	2.6	1.9	2.5	1.6	2.7	1.8	2.7	1.7	2.7	2.0	2.7	2.0
6	200	2.6	2.1	2.4	1.8	2.8	2.4	2.6	1.8	2.4	1.9	2.8	1.8	2.5	1.5	2.4	1.6	2.7	1.8	2.5	1.0
6	200	2.5	2.2	2.2	1.6	2.6	1.8	2.6	2.1	2.7	1.5	2.6	1.5	2.7	1.2	2.8	1.5	2.6	1.4	2.7	1.9
7	200	2.8	2.3	2.7	2.1	2.6	1.8	2.6	1.7	2.9	2.1	2.6	1.5	2.6	1.5	2.8	1.6	2.8	1.2	2.6	1.5
7	200	2.5	1.9	2.4	1.7	2.6	2.0	2.6	2.0	2.6	1.2	2.6	1.6	2.5	1.1	2.6	1.5	2.4	1.0	2.5	1.4
8	200	2.6	2.5	2.4	1.7	2.6	1.8	2.6	1.9	2.6	1.4	2.6	1.5	2.4	1.5	2.7	1.8	2.7	2.0	2.2	0.9
8	200	2.4	1.4	2.5	1.9	2.5	1.8	2.6	1.9	2.7	2.0	2.2	1.9	2.5	1.8	2.2	1.5	2.5	1.8	2.9	2.3
9	CTRL	2.3	1.7	2.7	2.1	2.7	2.1	2.6	1.7	2.7	2.1	2.8	1.8	2.7	1.7	2.7	1.7	2.4	1.7	2.6	1.8
9	CTRL	2.4	1.9	2.5	1.7	2.6	2.0	2.6	1.7	2.4	1.2	2.5	2.0	2.4	1.9	2.6	1.9	2.6	1.7	2.8	1.7
10	CTRL	2.5	1.8	2.5	1.9	2.6	2.0	2.6	1.7	2.5	1.2	2.4	1.3	2.6	1.5	2.9	2.1	2.3	1.4	2.6	1.5
10	CTRL	2.5	1.8	2.5	1.7	2.6	2.1	2.6	2.2	2.7	1.8	2.7	2.0	2.5	1.9	2.4	1.4	2.8	2.1	2.8	2.4
11	CTRL	2.7	2.1	2.5	1.8	2.7	1.7	2.6	1.5	2.8	1.6	2.5	1.1	2.5	1.3	2.7	1.3	2.7	1.7	2.4	1.6
11	CTRL	2.6	2.0	2.5	1.8	2.8	2.3	2.6	1.8	2.7	1.7	2.8	1.8	2.8	1.2	2.8	1.8	2.7	1.8	2.6	1.6
12	CTRL	2.5	1.8	2.5	1.9	2.4	2.2	2.6	1.9	2.5	1.7	2.3	1.6	2.6	1.7	2.6	2.1	2.6	2.1	2.8	1.8
12	CTRL	2.6	2.2	2.3	1.7	2.6	1.7	2.6	2.4	2.2	1.7	2.3	1.8	2.4	1.5	2.7	1.6	2.8	1.9	2.6	1.5
13	6	2.6	1.9	2.4	1.9	2.3	1.9	2.6	2.0	2.5	1.8	2.9	1.9	2.7	1.8	2.6	2.0	2.6	2.0	2.5	1.6
13	6	2.5	1.9	2.6	1.8	2.5	1.8	2.6	1.9	2.5	1.7	2.3	1.9	2.3	1.8	2.7	2.1	2.8	1.9	2.6	1.3
14	6	2.4	1.7	2.6	2.2	2.4	1.6	2.6	1.8	2.4	1.5	2.6	1.7	2.5	1.3	2.8	1.9	2.9	2.1	2.7	1.9
14	6	2.3	1.7	2.6	2.0	2.7	2.2	2.6	1.9	2.6	1.8	2.7	1.8	2.6	1.6	2.8	2.0	2.5	1.7	2.9	1.9
15	6	2.7	2.1	2.4	2.1	2.7	2.3	2.6	2.3	2.6	2.1	2.4	1.7	2.7	1.6	2.7	1.8	2.4	1.4	2.8	1.8
15	6	2.9	2.4	2.6	1.8	2.5	2.0	2.6	2.1	2.7	1.9	2.6	2.0	2.5	1.9	2.9	2.0	2.5	1.9	2.8	1.8
16	6	2.5	1.9	2.3	1.8	2.9	2.4	2.6	2.3	2.6	1.8	2.5	2.1	2.4	1.5	2.4	1.8	2.5	2.1	2.7	1.6
16	6	2.4	1.7	2.3	1.7	2.6	2.2	2.6	1.9	2.5	1.9	2.6	1.9	2.2	1.8	2.4	1.7	2.4	1.3	2.8	2.1
17	20	2.6	2.0	2.5	1.9	2.8	2.3	2.6	2.0	2.6	1.9	2.6	1.3	2.6	1.4	2.4	1.5	2.7	1.4	2.7	1.7
17	20	2.6	2.1	2.6	1.9	2.7	1.9	2.6	1.8	2.5	1.5	2.9	2.1	2.4	1.4	2.5	1.6	2.8	2.3	2.8	1.9
18	20	2.5	2.2	2.7	2.3	2.6	2.0	2.6	1.8	2.5	1.2	2.5	1.6	2.6	2.0	2.7	1.8	2.7	1.6	2.7	1.5
18	20	2.6	1.8	2.7	2.0	2.6	1.7	2.6	1.4	2.6	1.9	2.6	1.5	2.9	1.8	2.7	1.8	2.7	2.0	2.4	1.5
19	20	2.5	1.9	2.6	2.1	2.6	1.7	2.6	2.0	2.7	1.9	2.5	1.5	2.4	1.1	2.6	1.9	2.6	1.6	2.9	2.1
19	20	2.7	2.0	2.4	1.5	2.6	1.7	2.6	1.7	2.9	1.8	2.6	1.7	2.7	1.9	2.6	1.6	2.6	2.0	2.6	1.9
20	20	2.7	2.0	2.4	1.6	2.5	1.7	2.6	1.4	2.5	1.7	2.2	1.5	2.7	1.7	2.8	1.8	2.7	1.8	2.6	1.5
20	20	2.3	1.5	2.4	1.8	2.7	2.2	2.6	1.9	2.7	1.6	2.7	2.0	2.7	2.1	2.6	1.9	2.3	1.6	2.6	1.4
T°		24.4		25.3		25.2		24.9		24.7		25.4		25.7		25.1		25.7		24.7	24.7
H.R		56		52		55		56		51		54		54		51		48		52	57

Cage	**IMI** ng/100 ml	31	1	2	3	4	5	6	7	8	9	Total Mortality	N (end) 2010-07-26	N (beginning) 2010-07-16
1	60									1		1	30	31
2	60									1	1	2	33	35
3	60											0	36	36
4	60		1				2		1			4	31	35
5	200											0	35	35
6	200											0	35	35
7	200								1		1	2	33	35
8	200					1						1	34	35
9	CTRL											0	35	35
10	CTRL								1		1	2	30	32
11	CTRL								1		4	5	29	34
12	CTRL											0	35	35
13	6											0	34	34
14	6								2	2		4	31	35
15	6								1			1	34	35
16	6											0	35	35
17	20											0	35	35
18	20											0	34	34
19	20											0	35	35
20	20					1	1					2	34	36
	Total	0	1	0	0	2	3	0	7	4	7	24		

IMI (ng/100 ml)	Replicate -	Protein (mg/g tissue)	AChE activity (mDO/g prot/h)
CTRL	1	33.9	0.380
CTRL	2	32.2	0.231
CTRL	3	21.0	0.790
CTRL	4	22.5	0.728
CTRL	5	36.3	0.475
CTRL	6	37.2	0.336
CTRL	7	33.1	0.439
CTRL	8	40.6	0.375
CTRL	9	27.6	0.833
CTRL	10	37.3	0.468
6	1	28.0	0.471
6	2	25.9	0.617
6	3	37.8	0.533
6	4	34.1	0.348
6	5	33.0	0.204
6	6	40.4	0.442
6	7	38.4	0.334
6	8	42.4	0.645
6	9	33.0	0.380
6	10	48.7	0.278
20	1	20.0	0.946
20	2	18.9	0.793
20	3	29.4	0.540
20	4	39.4	0.290
20	5	25.5	0.334
20	6	32.0	0.469
20	7	26.2	0.393
20	8	44.7	0.415
20	9	29.3	0.529
20	11	39.5	0.346
60	1	22.0	0.764
60	2	10.9	2.223
60	3	29.9	0.356
60	4	35.6	0.317
60	5	38.0	0.733
60	6	53.6	0.614
60	7	41.5	0.381
60	8	38.2	0.338
60	9	42.8	0.297
60	10	36.2	0.777
200	1	36.8	0.223
200	2	26.2	0.535
200	3	24.1	0.527
200	4	27.5	0.494
200	5	21.9	0.674
200	6	37.7	0.364
200	7	42.2	0.342
200	8	35.4	0.359
200	9	33.4	0.379
200	10	46.9	0.430

IMI (ng/100ml)	Rep. -	Mass (g)	METs (ng/g. tiss.)	13-cis-RA (ng/g. tiss.)	9-cis-RA (ng/g. tiss.)	ROL (ng/g. tiss.)	RALD (ng/g. tiss.)
CTRL	1	1.422	8.64	6.45	12.50	24.63	39.85
CTRL	2	1.509	10.39	4.90	16.90	39.35	44.10
CTRL	3	1.435	7.26	4.58	9.02	27.73	ND
CTRL	4	1.513	4.54	4.86	7.34	28.70	33.51
CTRL	5	1.536	10.05	4.91	9.00	27.16	40.65
CTRL	6	1.408	5.52	4.40	13.34	ND	27.39
CTRL	7	1.368	ND	6.51	13.62	28.75	33.91
CTRL	8	1.449	8.96	4.62	7.74	35.09	54.64
6	1	1.464	4.71	4.94	11.49	ND	40.21
6	2	1.391	17.51	8.45	10.50	23.60	37.95
6	3	1.487	ND	6.25	15.96	18.34	ND
6	4	1.363	12.74	5.55	9.50	25.07	31.43
6	5	1.345	10.02	7.09	5.43	36.96	26.59
6	6	1.413	11.12	6.34	11.56	33.45	50.23
6	7	1.443	15.74	6.09	13.65	22.01	29.42
6	8	1.551	11.37	5.55	9.03	32.09	33.12
20	1	1.501	12.96	4.76	7.23	24.04	32.23
20	2	1.465	14.91	4.94	8.62	19.05	34.31
20	3	1.420	ND	4.14	16.24	23.10	36.85
20	4	1.366	6.33	4.34	8.64	22.15	39.80
20	5	1.439	13.46	5.10	13.65	ND	43.21
20	6	1.463	14.86	ND	9.68	21.47	24.19
20	7	1.486	15.92	5.97	11.85	18.91	24.71
20	8	1.380	1.78	6.34	10.65	22.02	37.81
60	1	1.611	12.41	ND	ND	ND	ND
60	2	1.503	ND	5.89	13.43	62.61	43.87
60	3	1.597	9.70	5.96	10.03	25.82	34.13
60	4	1.484	ND	ND	ND	ND	ND
60	5	1.452	4.86	5.83	13.87	30.60	33.55
60	6	1.522	ND	5.35	9.50	35.34	35.95
60	7	1.482	6.72	5.44	5.96	28.15	28.72
60	8	1.518	7.69	4.66	7.55	23.53	40.28
200	1	1.544	12.22	5.61	13.45	26.16	49.04
200	2	1.471	10.40	6.78	9.76	26.77	42.48
200	3	1.490	11.15	5.86	13.42	24.36	31.57
200	4	1.476	5.70	5.28	18.57	17.86	36.11
200	5	1.405	6.84	4.76	8.62	27.53	27.10
200	6	1.408	13.45	5.42	12.16	36.01	38.96
200	7	1.368	22.55	6.11	12.44	31.70	44.30
200	8	1.449	6.58	4.80	8.13	ND	33.45

ND = Non detectable

IMI (ng/100ml)	Rep. -	lutein (ng/g. tiss.)	zeaxanthin (ng/g. tiss.)	A-crypto. (ng/g. tiss.)	B-crypto. (ng/g. tiss.)	A-carot. (ng/g. tiss.)	B-carot. (ng/g. tiss.)
CTRL	1	170.78	151.67	103.52	68.43	246.44	98.13
CTRL	2	179.65	147.88	79.94	47.30	238.90	137.51
CTRL	3	167.08	144.53	89.05	70.24	249.19	165.33
CTRL	4	130.85	137.86	73.19	67.72	263.86	66.82
CTRL	5	171.80	130.69	85.08	73.67	235.81	187.78
CTRL	6	134.96	124.98	65.29	60.80	271.67	85.39
CTRL	7	192.51	173.86	109.24	81.73	264.06	71.23
CTRL	8	120.26	117.99	63.33	59.72	257.70	113.92
6	1	168.09	149.95	88.61	80.92	287.79	99.12
6	2	165.46	166.38	82.63	65.47	255.91	69.08
6	3	157.52	132.84	72.71	71.43	254.07	154.99
6	4	147.79	116.72	87.07	62.96	284.59	159.00
6	5	152.82	123.54	69.61	57.53	249.78	179.82
6	6	175.77	141.24	92.13	70.04	287.60	134.02
6	7	144.46	151.33	71.09	68.66	220.67	92.06
6	8	153.78	140.44	82.16	70.95	266.25	102.51
20	1	159.05	148.08	84.50	65.34	281.24	64.25
20	2	154.86	127.62	78.67	78.88	179.33	155.87
20	3	142.57	125.61	67.13	67.67	240.68	86.54
20	4	128.35	106.39	ND	59.98	227.85	148.07
20	5	134.13	139.82	71.94	73.85	299.93	81.97
20	6	197.05	160.57	84.48	73.30	249.32	190.82
20	7	163.98	145.11	70.15	73.26	269.81	90.95
20	8	172.11	133.93	63.00	55.45	257.92	147.41
60	1	ND	ND	ND	ND	ND	ND
60	2	143.09	130.86	69.83	53.03	198.84	70.74
60	3	133.38	97.15	68.02	53.10	248.87	118.79
60	4	ND	ND	ND	ND	ND	ND
60	5	160.08	135.71	89.96	63.50	ND	83.18
60	6	127.81	128.27	61.74	65.76	220.42	48.17
60	7	134.57	119.84	61.20	56.38	202.05	83.94
60	8	130.17	125.21	77.70	68.67	211.58	62.14
200	1	187.70	163.90	79.59	78.85	253.29	146.88
200	2	164.42	120.23	75.01	63.52	252.44	184.25
200	3	126.20	149.89	69.41	66.55	214.37	52.77
200	4	151.65	132.84	79.71	84.25	226.41	120.15
200	5	116.62	106.67	55.06	61.61	236.00	91.06
200	6	151.51	151.77	70.70	60.24	198.66	90.91
200	7	165.92	157.18	83.69	65.82	226.32	160.70
200	8	173.43	146.18	74.47	61.72	259.52	105.01

ND = Non detectable

IMI	Rep.	A-tocopherol	Triglycerides	TBARs
(ng/100ml)	-	(ng/g. tissue)	(ng/g tissue)	(ng/ug of trigly)
CTRL	1	166.23	475.3	0.684
CTRL	2	180.11	329.3	1.111
CTRL	3	185.72	525.4	0.649
CTRL	4	178.56	413.7	1.113
CTRL	5	168.60	567.6	0.550
CTRL	6	147.47	393.5	0.704
CTRL	7	210.19	637.8	0.647
CTRL	8	161.51	620.8	0.503
6	1	134.28	541.7	0.666
6	2	166.10	567.3	0.745
6	3	169.98	408.8	0.908
6	4	154.51	424.4	0.686
6	5	171.98	622.1	0.578
6	6	199.91	570.1	0.810
6	7	157.74	522.2	0.603
6	8	173.91	363.5	0.810
20	1	149.13	470.4	0.442
20	2	136.15	421.7	0.814
20	3	151.36	363.1	1.388
20	4	165.33	504.7	0.649
20	5	157.98	512.9	0.625
20	6	ND	487.4	0.880
20	7	150.63	394.9	1.066
20	8	165.15	372.9	0.945
60	1	ND	557.4	0.951
60	2	168.33	350.9	1.022
60	3	124.69	377.8	0.958
60	4	ND	541.9	0.279
60	5	178.76	526.4	0.329
60	6	155.75	645.7	0.633
60	7	132.99	405.5	1.039
60	8	127.49	483.8	0.493
200	1	190.66	370.9	1.114
200	2	195.73	733.3	0.366
200	3	140.56	450.1	0.349
200	4	124.16	514.7	0.650
200	5	129.84	465.9	0.889
200	6	151.13	507.8	0.439
200	7	173.55	574.7	0.306
200	8	160.64	460.3	0.814

ND = Non detectable
